# RT-CLAD: Artificial Intelligence-Based Real-Time Chironomid Larva Detection in Drinking Water Treatment Plants

**DOI:** 10.3390/s24010177

**Published:** 2023-12-28

**Authors:** Goeun Jang, Wooseong Yeo, Meeyoung Park, Yong-Gyun Park

**Affiliations:** 1 Department of Environmental and Energy Engineering, Chonnam National University, 77 Yongbong-ro, Buk-gu, Gwangju 61186, Republic of Korea; jge4954@gmail.com; 2 Department of Computer Engineering, Kyungnam University, 7 Gyeongnamdaehak-ro, Masanhappo-gu, Changwon-si 51767, Republic of Korea; 2022111831@student.kyungnam.ac.kr

**Keywords:** drinking water treatment plant, chironomid larvae, larval detection system, multi-spectral image, deep learning

## Abstract

The presence of chironomid larvae in tap water has sparked public concern regarding the water supply system in South Korea. Despite ongoing efforts to establish a safe water supply system, entirely preventing larval occurrences remains a significant challenge. Therefore, we developed a real-time chironomid larva detection system (RT-CLAD) based on deep learning technology, which was implemented in drinking water treatment plants. The acquisition of larval images was facilitated by a multi-spectral camera with a wide spectral range, enabling the capture of unique wavelet bands associated with larvae. Three state-of-the-art deep learning algorithms, namely the convolutional neural network (CNN), you only look once (YOLO), and residual neural network (ResNet), renowned for their exceptional performance in object detection tasks, were employed. Following a comparative analysis of these algorithms, the most accurate and rapid model was selected for RT-CLAD. To achieve the efficient and accurate detection of larvae, the original images were transformed into a specific wavelet format, followed by preprocessing to minimize data size. Consequently, the CNN, YOLO, and ResNet algorithms successfully detected larvae with 100% accuracy. In comparison to YOLO and ResNet, the CNN algorithm demonstrated greater efficiency because of its faster processing and simpler architecture. We anticipate that our RT-CLAD will address larva detection challenges in water treatment plants, thereby enhancing water supply security.

## 1. Introduction

Water treatment plants play a crucial role in ensuring the safety and potability of water by eliminating impurities, pathogens, and chemicals from raw water. These facilities employ various processes, including water intake, coagulation and sedimentation, filtration, and disinfection [1]. In recent times, chironomid larva outbreaks have become a frequent concern in countries such as South Korea, United States, Scotland, and South Africa [2]. Consequently, it is imperative to gain a precise understanding of the occurrence of chironomid larvae in water treatment plants and actively explore appropriate technologies and methods for their monitoring and control [3].

Chironomid larvae, recently discovered in South Korea, are benthic macroinvertebrates commonly used as key organisms in freshwater ecosystem health assessments. Recognized as indicator species, these larvae are sensitive to various environmental changes and exposure to harmful substances [4]. Therefore, it is imperative to control their emergence and reproduction during water treatment processes [5].

In response to tap water larva outbreaks, the South Korean government conducted emergency inspections of water treatment plants and developed a comprehensive management plan. In addition, they incorporated artificial intelligence (AI) and operations and maintenance (O&M) technologies, including a telemonitoring system (TMS), to enable the accurate real-time monitoring of plant operations [6,7,8]. Despite the difficulty in completely preventing larval outbreaks, the increasing importance of a real-time larval detection monitoring system based on artificial intelligence (AI) has become evident. This approach holds promise for effectively monitoring and managing such incidents.

Therefore, our study aimed to determine the real-time presence or absence of larvae in water treatment plants using AI algorithms. To achieve this, we propose a real-time chironomid larva detection system (RT-CLAD), which combines multispectral imaging with advanced deep learning models, with the aim of rapid and accurate larval detection. We conducted a comparative analysis of three widely recognized deep learning algorithms for image classification: the convolutional neural network (CNN), pre-trained residual neural network (ResNet), and object detection methods such as the you only look once (YOLO) algorithm. The objective of this study was to identify an optimal model for detecting chironomid larvae in drinking water treatment plants. Multispectral imaging technology is employed to detect and classify material changes in water treatment plants by acquiring spectral and spatial information on specific objects across multiple wavelengths, facilitating the rapid determination of larvae. By recording information about an object, multispectral imaging detects even the smallest objects invisible to the naked eye, providing accurate and detailed information [9]. Our contributions and novelties are as follows: (1) employing a multispectral camera to obtain real-time images inside water treatment plants; (2) implementing an image preprocessing pipeline to isolate the specific wavelengths associated with larvae; and (3) developing a real-time larva detection algorithm based on AI.

Consequently, in contrast to pretrained complex image classification and object detection algorithms such as ResNet and YOLO, CNN has demonstrated superior performance in terms of speed and accuracy. Our comparative assessment of the AI algorithms has enabled the successful development of an optimized system for real-time larval detection.

## 2. Materials and Methods

### 2.1. Overview of the Real-Time Chironomid Larva Detection System (RT-CLAD)

RT-CLAD is designed to accurately detect chironomid larvae in real-time. Our proposed system incorporates data preprocessing to efficiently reduce the input data size and enhance algorithm efficiency. In addition, it is designed to select the most effective algorithm for larva detection among various outstanding deep learning algorithms, prioritizing efficiency over complex deep learning structures.

**Figure 1:**
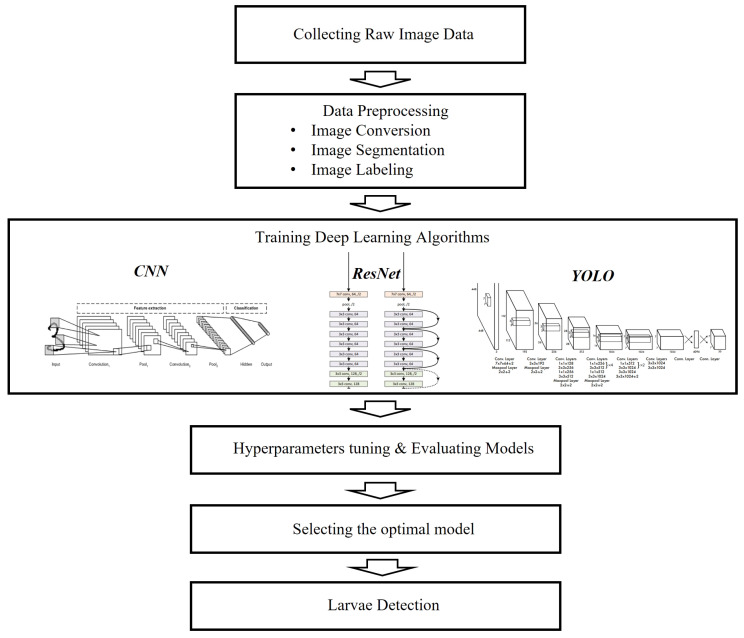
Analysis workflow for the real-time chironomid larval detection system (RT-CLAD). Raw images were initially collected and subsequently preprocessed as inputs for CNN, YOLO, and ResNet deep-learning models. The selection of the optimal model was based on the evaluation of various metrics, and this chosen model will be employed for larval detection.

To evaluate the relative detection accuracy of chironomid larvae using RT-CLAD, the study was extended to similar-looking species such as yellow worms and mosquito larvae. The workflow for RT-CLAD is outlined as follows: we captured 200 images of each—chironomid larvae, yellow worms, and mosquito larvae—using a multispectral camera. Subsequently, these images were converted into wavelet files based on the following wavelength bands: green (GRE), near-infrared (NIR), red (RED), and red edge (REG). Given that the green (GRE) band was the most effective for visually representing the three larval types, we selected 200 wavelet-transformed files from the GRE band for each larval type as input for our deep-learning models. The training of RT-CLAD involved the use of multiple deep learning models, namely CNN, YOLO, and ResNet, to determine the most optimal model. In the process of identifying the optimal model, we assessed their detection performance using metrics such as accuracy, precision, recall, and F1-score. The comprehensive analysis workflow of RT-CLAD is illustrated in Figure 1.

### 2.2. Chironomid Larvae

**Figure 2:**
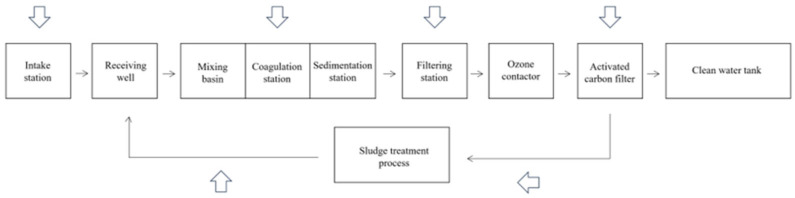
Process of an advanced drinking water treatment plant.

Chironomid larvae, classified as benthic macroinvertebrates, are the most abundant members of the fly family in aquatic ecosystems. They inhabit various ecosystems, including lakes, oceans, and rivers. Notably, in Korean river ecosystems, the stonefly family, to which they belong, accounts for over 50% of invertebrates [10]. With the ability to achieve densities of 100,000 individuals per cubic meter of water, these larvae can thrive in depths of up to 1000 m and endure temperatures as low as minus 16 °C. Their life cycle encompasses four stages, spanning from egg to adulthood, with durations ranging from three weeks to several months [11]. The lifespans of larvae can range from two weeks to four years depending on the species [12]. These larvae enter drinking water treatment plants and are delivered to the tap water without being completely removed. Figure 2 shows the locations where the larvae occur in the drinking water treatment process, with arrows indicating the routes of their entry.

### 2.3. Multispectral Camera

**Figure 3:**
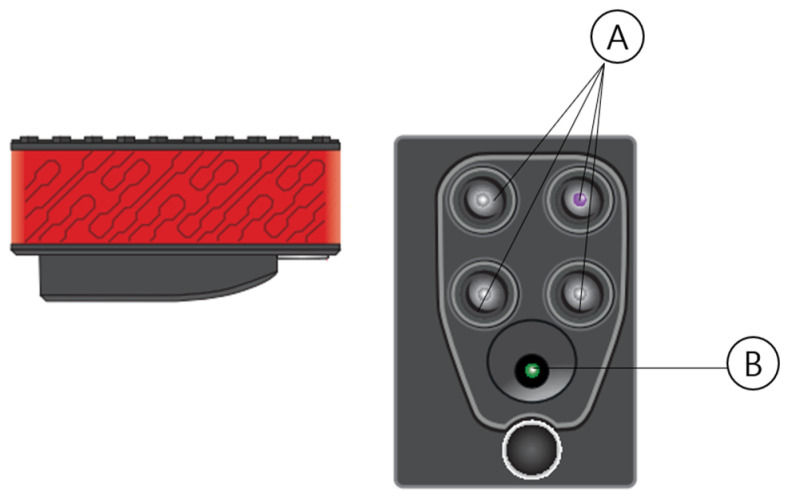
Multispectral camera. Megapixel monochrome sensors collect data in discrete spectral bands: (**A**) multispectral imaging sensors operating in green (550–40 nm), red (660–40 nm), red edge (735–10 nm), and near-infrared (790–40 nm). (**B**)—Megapixel RGB sensor.

The multispectral camera used in this study was Parrot’s Sequoia (Parrot, Paris, France), weighing 72 g and measuring 5.9 cm × 4.1 cm × 2.8 cm. It can capture spectral images across five bands, including the visible light region. It operates in the GRE band with a wavelength of 550 nm and a bandwidth of 40 nm, RED band with a wavelength of 660 nm and a bandwidth of 40 nm, REG band with a wavelength of 735 nm and a bandwidth of 10 nm, and NIR band with a wavelength of 790 nm and a bandwidth of 40 nm. The multispectral camera is shown in Figure 3. The captured images can be analyzed using various software.

### 2.4. Convolutional Neural Networks (CNNs)

Image classification algorithms, such as CNNs, are designed to classify labels for specific objects within an image. CNN, a deep learning-based neural network model, excels in recognizing and classifying objects in image and video processing [13]. Its structure includes convolutional, pooling, and fully connected layers, with particular significance attributed to the convolutional layer due to its locally connected weights and features. These layers function together, with increasing channels and decreasing dimensions, to effectively extract and learn features for object detection [14,15,16]. For the purpose of this study, which focused on detecting larvae in water treatment plants, the primary goal was to determine the real-time presence or absence of larvae. Therefore, in this study, we opted for a CNN with simpler structures over more complex models to achieve accurate object detection with reduced computational complexity.

### 2.5. Pre-Trained Deep Learning Algorithms

We import a pre-trained Resnet101 model from ImageNet to extract the features. In traditional CNN structures, deep layer creation leads to gradient disappearance when a certain depth is reached, causing suboptimal or sluggish learning, revealing the limitations of deep learning performance [17]. To address these problems, ResNet, which is based on the VGGNet structure and has a 152-layer network structure that is eight times deeper [18], was introduced. ResNet stands out for its enhanced performance, consistent parameter count, and reduced training time, which are achieved through efficient computing both within and between layers [19]. ResNet achieved first place in the ImageNet Large Scale Visual Recognition Challenge (ILSVRC) 2015 [17,20]. By overcoming gradient vanishing through residuals, ResNet achieves increased accuracy as layers become deeper, positioning it as a more complex structure among CNN-based architectures such as AlexNet, GoogLeNet, VGG, and DenseNet. In contrast, our focus was on contrasting this complexity with the straightforward structure of CNN. Notably, both CNN and ResNet were effective in detecting larvae in our study.

However, other pre-trained object detection algorithms, such as YOLO, not only identify objects but also provide their location within the image. This feature is crucial in tasks such as counting similar objects and distinguishing among various items [21]. YOLO is a unified object model that does not require a separate network to extract candidate regions, which makes it simple to configure and suitable for real-world applications by directly training the entire image [22]. Notably, it excels in real-time object detection in terms of processing time [23].

YOLO has undergone consistent updates, evolving from version 1 to 8, progressively enhancing both speed and accuracy. Chien-Yao Wang et al. introduced the YOLOv7 model used in our study and demonstrated a validation average precision (Val AP) of 51.2%, surpassing the 50.7% achieved by the YOLOv5-X model trained on the COCO 2017 dataset [24]. YOLOv8, being a more recent model, exhibits superior performance to v7. However, it lacks support for high-resolution (1280) images and sufficient related data. Furthermore, employing it for real-time detection may result in unexpected errors. Therefore, YOLOv7 was chosen for its commendable performance and stability. In addition, it is a faster and more accurate algorithm than other real-time object detection model [25].

## 3. Results

### 3.1. Multispectral Images

**Figure 4:**
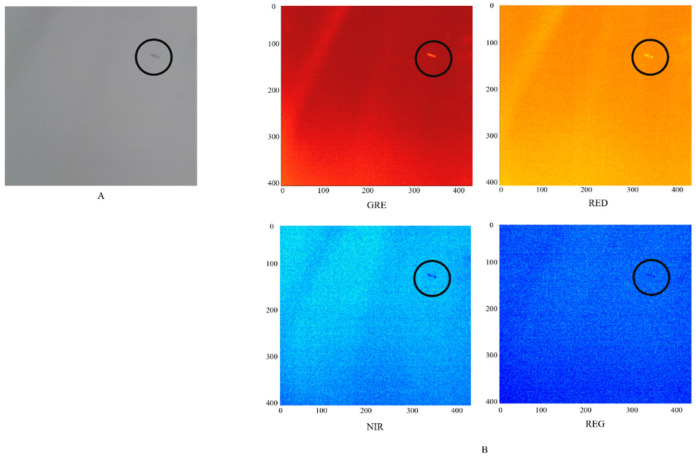
Result of photographing a red chironomid larva with a multispectral camera. (**A**) Original image. (**B**) Multispectral image: green (550–40 nm), red (660–40 nm), red edge (735–10 nm), and near-infrared (790–40 nm). The circle indicates the location of the larvae.

Multispectral images of the collected red chironomid larvae were captured using a camera. The larvae were placed on a Teflon plate with 99% light absorption, maintaining a fixed distance of 30 cm between the device and the object. The resulting images are shown in Figure 4. General natural light illuminated the environment during photography. The wavelet conversion of the original photo revealed that the GRE band was the most recognizable, indicating that the multispectral image converted to the GRE wavelength band is a suitable band for larval detection.

### 3.2. Analysis Results of Multispectral Image

**Figure 5:**
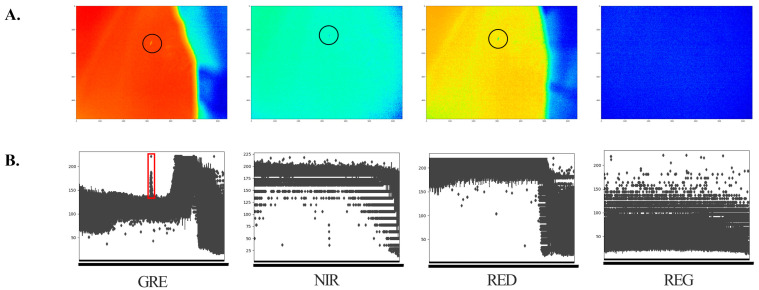
Data distribution of wavelet images by spectral bands. (**A**) Wavelet-transformed images for GRE, NIR, RED, and REG bands. The circle indicates the location of the larvae. (**B**) Boxplot of the wavelet-transformed image file. The target was observed in terms of the density and width of specific values compared to the background and noise regions. The region highlighted by the red box is the larval image.

The original image resolution was 1200 × 1200, with the typical larvae observation area being less than 100 × 100. Processing the detection target for a deep learning model in such images is challenging because of its significant size. In addition, noise was introduced during the conversion of the original files to the wavelet format. To determine the input data for deep learning, we created box plots to examine the data distribution (Figure 5). In contrast to other bands, larvae appeared in a narrow and elongated column shape in the GRE region. Therefore, the GRE region was chosen as the input data. As shown in Figure 5, the wavelength of larvae in the image was narrow and sharp in comparison to the background and noise.

Images contaminated with noise could pose a significant challenge for real-time detection, potentially impeding the learning process of the model. To address this challenge, image segmentation was conducted to reduce noise. This involved cropping the images to a predefined size and subsequently positioning the larvae in the center.

### 3.3. Image Segmentation

**Figure 6:**
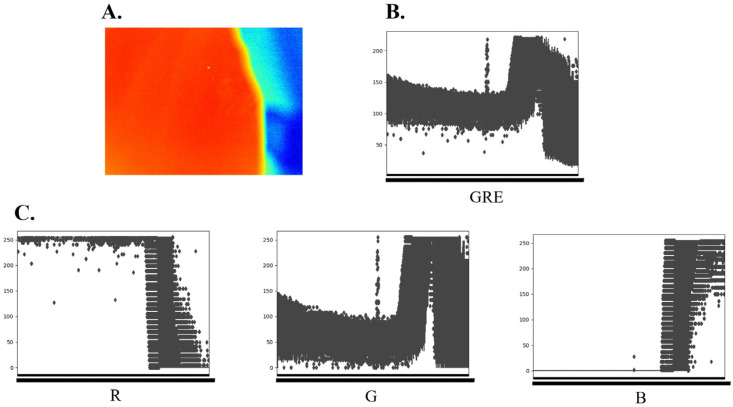
Original image and RGB-separated images. (**A**) Original GRE image. (**B**) Box plot for the original GRE region. (**C**) Box plots for red (R), green (G), and blue (B) channels. G displays a vertically expanded graph derived from the original GRE band. This enhances differentiation between the background and larvae.

Initially, efforts were made to identify the coordinates of the narrow and elongated columns in the GRE box plot for segmentation. However, in files heavily affected by noise, pinpointing the larvae’s location on the box plot proved impossible. To address these limitations, we conducted data analysis using RGB separation in color images. The box plot characteristics of the R, G, and B components separated from the GRE image are illustrated in Figure 6. The G component exhibits a vertically expanded graph derived from the original GRE band, resulting in a broader data distribution than the original image. This expansion enhances the distinction between the background and larvae. The data distribution of the R and B graphs exhibited a reflection along the x-axis, with no other distinguishing characteristics. To achieve algorithm generalization, we employed G as the image input data, representing larvae with high pixel values.

By identifying the maximum coordinate values on the graph, it is possible to determine the larval coordinates for all data. We applied the coordinates obtained in the G component to the original image with channels not separated, resulting in the cropping of larvae at a specific size. The image size was standardized to 300 × 300 to ensure the sufficient inclusion of larvae of any size. Segmentation offers several advantages. First, the 300 × 300 images obtained after segmentation have reduced file sizes compared with the original images, leading to decreased training times. Furthermore, segmentation eliminates unnecessary areas from the images, increasing the proportions of the images occupied by larvae. This facilitates the faster convergence of deep learning algorithms, excluding irrelevant regions from the learning process.

### 3.4. Data Augmentation

After image segmentation, we obtained 169 images of chironomid larvae, 128 images of yellow worms, and 94 images of mosquito larvae. Training a deep learning model solely on the prepared 100–200 images may be insufficient for capturing the target’s patterns. Hence, we created new data by applying augmentation techniques to the four-times dataset, involving random rotations, horizontal and vertical shifts, horizontal flipping, and zooming in and out. Augmentation effectively addressed the shortage of original data by significantly increasing the dataset size, which is advantageous for training a deep learning model. A summary of the augmented data is presented in Table 1.

### 3.5. Deep Learning Model Training

To train and evaluate the performance of both binary classification and object detection models, we divided the input data into training and test datasets using a 7:3 ratio. The CNN model consists of 10 layers whereas the Resnet101 model comprises 314 layers. Table 2 presents the architecture of each model, excluding YOLOv7. This exclusion is due to YOLOv7 being a pre-trained and highly complex model, which makes its detailed architectural representation beyond the scope of this table.

### 3.6. Hyperparameters Tuning

Through experimentation, a batch size of 32 was found to be suitable for training the deep learning model. Larger batch sizes proved unstable during training and improved performance compared with the batch size of 32. To identify a suitable optimizer for the input data, we compared Adam, SGD, and RMSprop, which are effective for binary classification tasks [20]. SGD exhibited the slowest accuracy improvement and unstable training. RMSprop showed a faster increase in accuracy but still performed with training instability compared with SGD. Adam demonstrated the most stable training and rapidly converged to an accuracy of 1.0. Consequently, Adam was chosen as the optimizer for CNN and Resnet101. For YOLOv7, which allowed for both Adam and SGD, we selected SGD because of its superior performance. Regarding the number of epochs, the model converged within 300 epochs, and exceeding that resulted in performance degradation due to overfitting. Therefore, to identify the optimal hyperparameters, tuning was conducted with 200 and 300 epochs. Subsequently, we tuned the learning rate. For the Adam optimizer, high learning rates, such as 0.05 or 0.1, resulted in either divergence or oscillation during the training process. Because the model converged with a learning rate of at least 0.01, we tuned the learning rate using values of 0.00001, 0.0001, 0.001, and 0.01. For YOLOv7, the learning rate was dynamically adjusted through OneCycleLR, eliminating the need for separate tuning.

### 3.7. Deep Learning Model Performance

**Figure 7:**
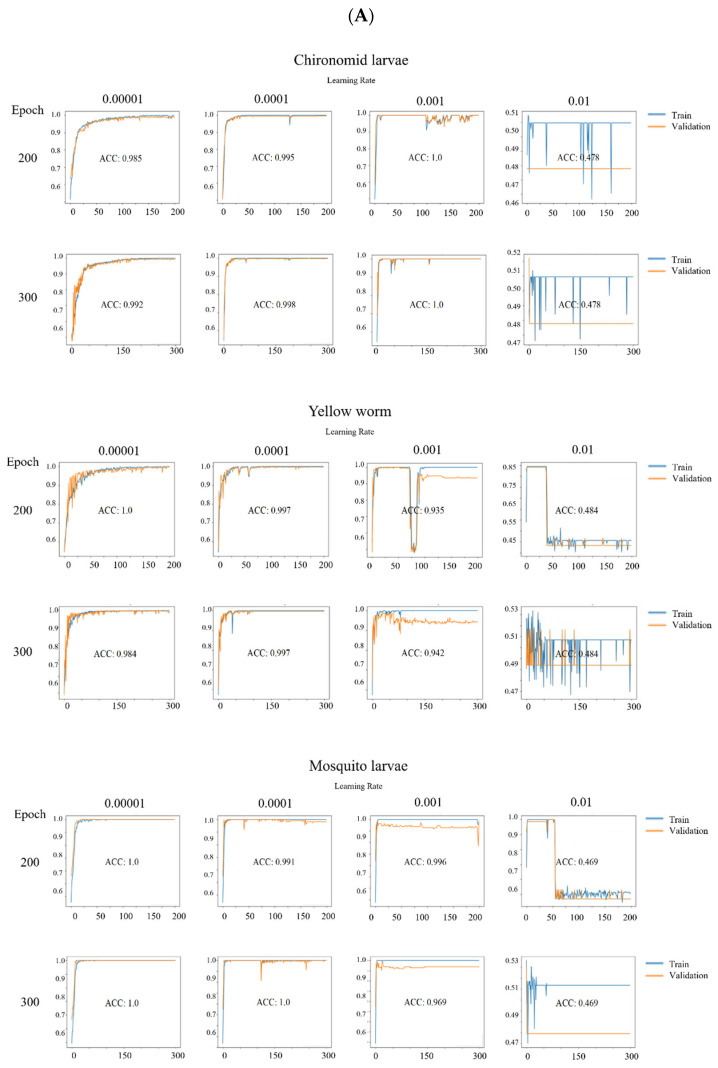

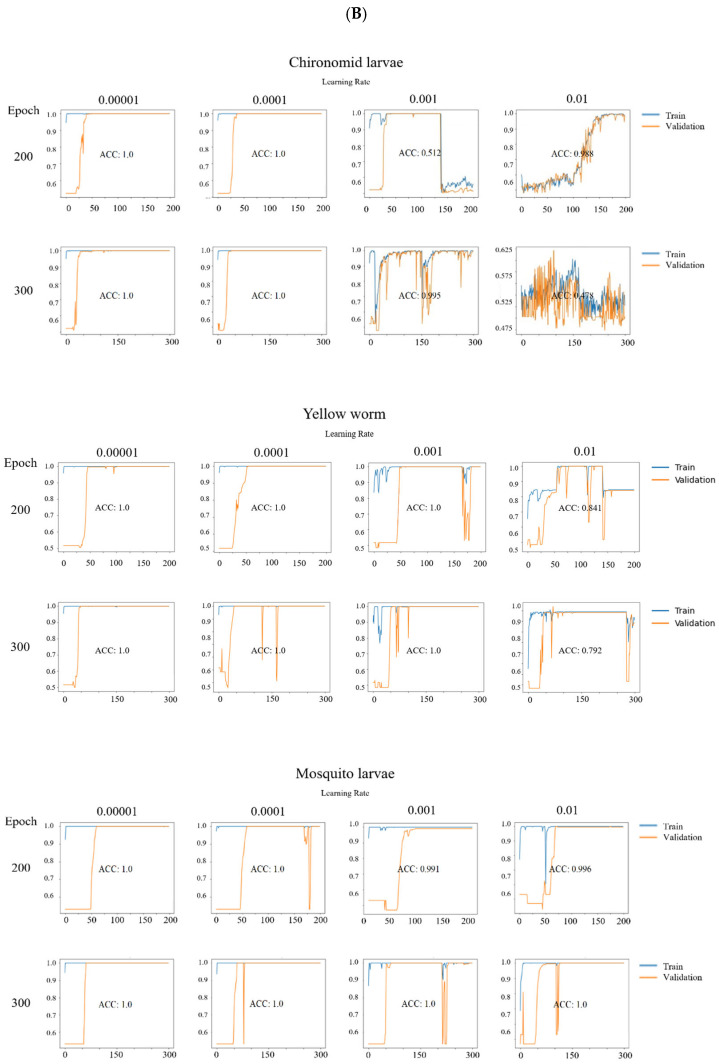

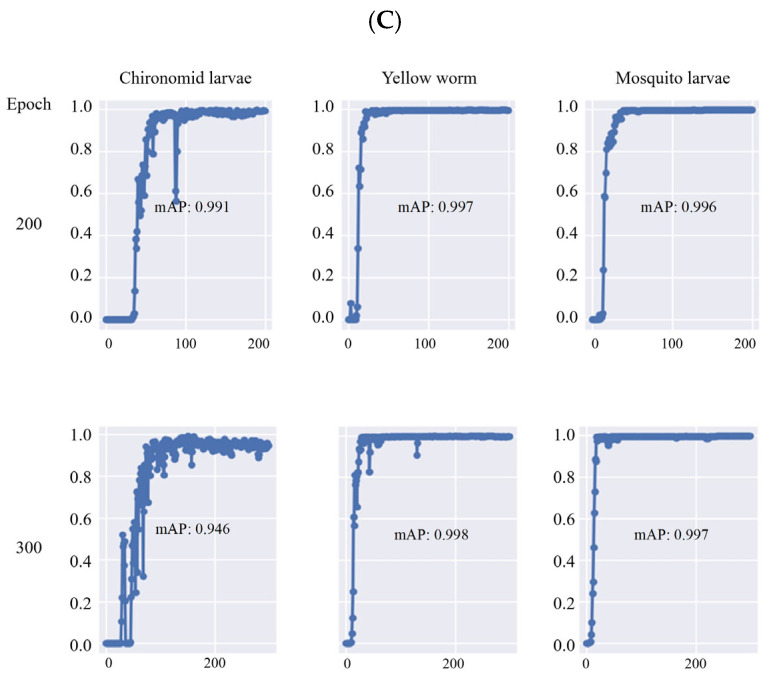
Performance results: accuracy and loss graph of each model for chironomid larvae, yellow worms, and mosquito larvae. (**A**) CNN performance results. (**B**) Resnet101 results. (**C**) Yolov7 results. ACC: Accuracy; mAP: mean average precision.

Hyperparameter tuning was performed for eight combinations of epochs (200, 300) and learning rates (0.00001, 0.0001, 0.001, 0.01) (Figure 7). The optimal model was determined based on the highest accuracy and the lowest loss among these eight models. For chironomid larvae, the optimal model was found to be Epoch 300, with a learning rate of 0.001. For the yellow worm, the optimal model was identified to be Epoch 200, with a learning rate of 0.00001. Finally, for mosquito larvae, the optimal model was identified as Epoch 300, with a learning rate of 0.00001. The experimental environment used in this study is described in Table 3.

### 3.8. Optimal Model Selection

The performance of the deep learning models was evaluated using the accuracy and loss functions. YOLOv7 uses mAP, a crucial metric in object detection tasks that provides a comprehensive assessment of a model’s ability to detect and localize objects across various classes within an image. In addition, confusion matrices were computed by comparing the actual and predicted label values. All algorithms demonstrated a 100% accuracy rate with the validation data. The primary focus of our paper is real-time larval detection, and thus, we aimed to identify the fastest and most accurate algorithm. In real-time larval detection, higher model accuracy is indicated by a lower error between the predicted probabilities for larval images and the actual label values. Furthermore, models with shorter training times can promptly adapt to newly collected training data. Therefore, the root mean squared error (RMSE) and speed were used as new evaluation metrics for model selection (Table 4). The speed of CNN and Resnet101 was determined by measuring the time interval between the start and end of the training process. For YOLOv7, the speed was calculated by converting the time value generated after training into seconds. Based on the larval detection evaluation metrics of speed and RMSE, we reported that CNN is the most suitable algorithm for real-time larval detection.

The performance of the models was evaluated for three classes (chironomid larvae, yellow worms, and mosquito larvae). Resnet101 exhibited the best performance in terms of RMSE but had the slowest speed, making it impractical for rapidly detecting new data. YOLOv7 had a decent speed but the highest RMSE, leading to reduced prediction accuracy. CNN, while having a higher RMSE than Resnet101, had the fastest speed, ensuring reasonable prediction reliability while allowing for rapid updates in real-time environments.

## 4. Conclusions

This study proposes RT-CLAD, a real-time larva detection system that integrates multispectral imaging technology with deep learning algorithms, with the aim of achieving the accurate and rapid detection of larvae in drinking water treatment plants. Spectral images across five wavelength bands, including the visible light spectrum, were acquired for each type of larva. We used the wavelet transform file of the GRE band, which was identified as effective for larval detection. Multispectral images were processed through image segmentation and color separation to minimize noise and contamination. This preprocessing step heightened larval presence in the images, thereby accelerating the learning speed of the algorithm.

Subsequently, we used deep learning algorithms such as CNN, Resnet101, and YOLOv7 for larval identification. All algorithms demonstrated a 100% accuracy rate with the validation data. The primary focus of our study was real-time larval detection; thus, we sought to identify the fastest algorithm. We reported that based on larval detection evaluation metrics of speed and RMSE, CNN is most suitable for real-time larval detection in drinking water treatment plants.

Despite its merits, our study has notable limitations. First, the crucial consideration of light sources for capturing images using the multispectral camera was overlooked. Second, the scope of this study did not encompass the number of larvae in the water treatment system. To address these limitations and explore the impacts of varying light conditions and larval populations on the system’s efficacy, further research is required. In conclusion, our proposed system has potential applications in smart water treatment facilities, contributing to reliable and clean water supply and effective water quality management. Future research will be required to refine RT-CLAD by conducting experiments under various conditions such as different numbers of larvae, light sources, and interfering substances.

## Figures and Tables

**Table 1:** Total number of datasets.

	Chironomid Larvae	Yellow Worms	Mosquito Larvae
Raw	199	199	200
Segmented	169	128	94
Augmentation	676	512	376
Total image	676	512	376

**Table 2:** Architectures of deep learning models.

**2-1. CNN architecture**		
**Layer Type**	**Output**	**Description**
Conv2D	298 × 298	16 filters of size 3 × 3, ReLU activation
MaxPooling	149 × 149	2 × 2 Max Pooling
Conv2D	147 × 147	32 filters of size 3 × 3, ReLU activation
MaxPooling	73 × 73	2 × 2 Max Pooling
Conv2D	71 × 71	64 filters of size 3 × 3, ReLU activation
MaxPooling	35 × 35	2 × 2 Max Pooling
Flatten	78,400	Flattens the data
Dense	256 or 512	256 or 512 neurons, ReLU activation
Dropout	256 or 512	Dropout with 50% probability
Dense	1	1 neuron, Sigmoid activation
**2-2. Resnet101 architecture**		
**Layer Type**	**Output**	**Description**
Resnet101	300 × 300	Residual network, Transfer learning
Flatten	204,800	Flattens the data
Dense	256 or 512	256 or 512 neurons, ReLU activation
Dropout	256 or 512	Dropout with 50% probability

**Table 3:** The experimental environment.

Environment	Specification
Processor	12th Gen Intel^®^ Core™ i9-12900K, operating at 3.20 GHz
Memory	64 GB RAM
Graphics Card	NVIDIA GeForce RTX 3090 with 24 GB VRAM
CUDA	11.2.2
cuDNN *	8.1.1
Python	3.10.12 or 3.9.18
SW Packages	Tensorflow 2.10.0, Torch 1.7.1 + cu110, Torchvision 0.8.2 + cu110, OpenCV 4.6.0, Pillow 9.4.0, NumPy 1.25.2, Pandas 2.0.3, Matplotlib 3.7.2, Seaborn 0.12.2

* cuDNN: CUDA deep neural network.

**Table 4:** Comparison of RMSE and speed performance.

	Chironomid Larvae			Yellow Worms			Mosquito Larvae		
Model	CNN	Resnet101	Yolov7	CNN	Resnet101	Yolov7	CNN	Resnet101	Yolov7
RMSE	3.16 × 10^−0..5^	6.15 × 10^−11^	1.89 × 10^−0.1^	4.89 × 10^−0.2^	4.59 × 10^−19^	1.23 × 10^−0.1^	3.39 × 10^−0.3^	1.15 × 10^−13^	9.59 × 10^−0.2^
Speed (s)	883	3070	1029	485	1583	1292	743	1739	1033

## Data Availability

Data is contained within the article.
